# MicroRNA-155-5p Overexpression in Peripheral Blood Mononuclear Cells of Chronic Lymphocytic Leukemia Patients Is a Novel, Independent Molecular Biomarker of Poor Prognosis

**DOI:** 10.1155/2017/2046545

**Published:** 2017-12-31

**Authors:** Sotirios G. Papageorgiou, Christos K. Kontos, Marios A. Diamantopoulos, Anthi Bouchla, Eirini Glezou, Efthymia Bazani, Vasiliki Pappa, Andreas Scorilas

**Affiliations:** ^1^Second Department of Internal Medicine and Research Unit, University General Hospital “Attikon”, 1 Rimini St., Haidari, 12462 Athens, Greece; ^2^Department of Biochemistry and Molecular Biology, National and Kapodistrian University of Athens, Panepistimiopolis, 15701 Athens, Greece

## Abstract

MicroRNA-155-5p (miR-155-5p) is a proinflammatory, oncogenic miRNA, involved in various physiological processes, including hematopoiesis, immunity, inflammation, and cell lineage differentiation. It regulates important transcription factors, such as E2F2, hypoxia-inducible factor 1 (HIF1), and FOXO3. Recently, the dysregulation of miR-155-5p expression has been linked to chronic lymphocytic leukemia (CLL) pathogenesis. In this research study, we investigated the potential diagnostic and prognostic value of miR-155-5p in CLL. To achieve our goal, we isolated total RNA from peripheral blood mononuclear cells (PBMCs) collected from 88 CLL patients and 36 nonleukemic blood donors and performed polyadenylation of total RNA and reverse transcription. Next, we quantified miR-155-5p levels using an in-house-developed real-time quantitative PCR method, before proceeding to extensive biostatistical analysis. Thus, it appears that miR-155-5p is significantly overexpressed in PBMCs of CLL patients and can distinguish them from nonleukemic population. Kaplan-Meier OS analysis and bootstrap univariate Cox regression showed that high miR-155-5p expression predicts inferior OS for CLL patients (*p* < 0.001). Interestingly, miR-155-5p overexpression retains its unfavorable prognostic role in CLL patients stratified according to established prognostic factors [CD38 expression and mutational status of the immunoglobulin heavy chain variable region (*IGHV*)]. Thus, miR-155-5p appears as a promising, independent molecular biomarker of unfavorable prognosis in CLL.

## 1. Introduction

Chronic lymphocytic leukemia (CLL), the most common type of leukemia in adults, remains incurable, although patients' survival has been extended with discovery and implementation of new treatment options [1, 2]. The features of CLL comprise the progressive accumulation of small mature CD5^+^ B lymphocytes in the peripheral blood, lymphoid organs, and bone marrow [3]. The clinical course of its hematological malignancy is remarkably heterogeneous among patients [2]; most of them are elderly people. The average age at the time of CLL diagnosis is about 71 years, while it is rarely encountered in people under the age of 40 and is very uncommon in children [1].

Currently, two systems of disease classification are routinely used to predict the prognosis of CLL patients, namely, the Rai [4] and the Binet staging [5] systems. Though they may be useful, both staging systems do not predict therapy failure. Moreover, they are unable to discriminate between patients with probably evolving disease and those achieving stable disease for a long life period [6]. The prognosis of CLL patients is still based on clinicopathological finding; however, extensive research in the field during the last decade has unleashed the prospect of adding new prognostic biomarkers for CLL in the clinical routine [7], such as cytogenetic markers, mutational status of the immunoglobulin heavy chain variable region (*IGHV*), CD38 expression, and zeta-chain-associated protein kinase 70 kDa (ZAP70) expression in leukemic B cells [8, 9]. In this regard, partial deletion of chromosome 13 (not combined with other karyotype abnormalities), low percentage of ZAP70-positive and/or CD38-positive leukemic B cells (20% and/or 30%, resp.), and a mutated *IGHV* locus are indicative of favorable prognosis in CLL [10].

MicroRNAs (miRNAs) are small single-stranded noncoding RNA molecules that epigenetically regulate gene expression, mostly through binding to the 3′ untranslated region (3′-UTR) of targeted messenger RNA (mRNA) molecules and, hence, mediating translational repression, usually along with mRNA degradation too [11]. It is estimated that the human genome can produce more than a thousand of different miRNAs, which regulate approximately 60% of protein-coding genes, thereby controlling the activity and function of key signaling pathways and cellular processes such as cell proliferation, apoptosis, cell differentiation, and response to hypoxia [12]. In fact, miRNAs can function as either oncogenes or tumor suppressors, and some of them—including miR-15a, miR-16, miR-181b, and members of the miR-17/92 cluster—play pivotal roles in CLL onset and progression [13]. From a clinical perspective, particular miRNAs can also serve as biomarkers for CLL onset [14].

miR-155-5p is a proinflammatory, oncogenic miRNA, highly expressed in activated B and T cells as well as in macrophages. It is involved in various physiological processes, including hematopoiesis, immunity, inflammation, and cell lineage differentiation. Targets of miR-155-5p include several cytosolic and nuclear proteins that control the development of regulatory T cells [15]. For instance, miR-155-5p targets E2F2 transcription factor to enhance the angiogenic capability of induced endothelial cells [16]. Another target of miR-155-5p is hypoxia-inducible factor 1 alpha subunit (HIF1A), namely, the regulatory *α*-subunit of the heterodimeric HIF1 transcription factor [17]. HIF1 is a major effector of hypoxia, as it regulates many genes involved in cellular proliferation, migration, energy metabolism, angiogenesis, and apoptosis [18, 19]. Moreover, HIF1 is the transcription factor primarily responsible for miR-155-5p induction in hypoxia [17]. This oncomiR shows upregulated expression levels in several malignant solid tumors [20–22]. Aberrant expression of miR-155-5p has also been observed in diffuse large B cell lymphoma, acute myeloid leukemia, and CLL [23, 24]. Moreover, transgenic mice with B cells overexpressing miR-155-5p are very likely to develop B cell lymphomas [25].

The aforementioned evidence prompted us to assess the prognostic and diagnostic potential of miR-155-5p in CLL and to evaluate its putative clinical application as a molecular biomarker in this hematological malignancy. To achieve this goal, we developed an accurate and cost-effective quantitative real-time PCR (qPCR) methodology based on the SYBR® Green chemistry, for the relative quantification of miR-155-5p levels in leukemic B cells of CLL patients and PBMCs of normal blood donors.

## 2. Materials and Methods

### 2.1. Patients and Control Group

This original research study included 88 patients who had previously been diagnosed with CLL at the Second Department of Internal Medicine and Research Unit in University General Hospital “Attikon” (Athens, Greece) as well as 36 normal controls. None of the patients had received any treatment during the last six months before blood sample collection. Peripheral blood mononuclear cells (PBMCs) were positively selected after centrifugation on a Ficoll-Hypaque gradient. Most PBMCs isolated from patients' blood samples were leukemic B cells, as confirmed by cell immunophenotyping. The clinical stage of CLL was determined based on the Binet and Rai staging systems. Patients with Rai stage 0 CLL were considered to have a low risk, patients of stage I and II were considered to have an intermediate risk, and stage III and IV patients were classified in the high-risk group.

This research study was approved by the institutional Ethics Committee of the University General Hospital “Attikon” (Athens, Greece) and conducted in respect to the ethical standards of the Helsinki Declaration of 1964, as revised in 1983. A written informed consent was also signed from each participant of the study.

### 2.2. Cell Line Culture

The human histiocytic lymphoma cell line U-937 was propagated appropriately in Dulbecco's modified Eagle's medium, adjusted to contain 10% fetal bovine serum, 100 kU/L penicillin, 0.1 g/L streptomycin, and 2 mM/L glutamine. Cells were seeded at a concentration of 0.5 × 10^5^ cells/mL and incubated for 48 h at 37°C, in a humidified atmosphere containing 5% CO_2_, before being collected for further use.

### 2.3. Cell Immunophenotyping

Immunophenotyping of CLL cells was performed with a routine panel of evaluated monoclonal antibodies (Supplementary Materials and Methods is available here). Based on a previously established threshold [9], cases with more than 30% of CD38-expressing cells were characterized as positive for this marker, as previously described [26].

### 2.4. Nucleic Acid Extraction, RNA Polyadenylation, and Reverse Transcription of Poly(A) RNA

DNA and total RNA were extracted from isolated PBMCs and U-937 cells using the TRI Reagent® (Molecular Research Center Inc., Cincinnati, OH). The concentration and purity of purified total RNA were assessed with a BioSpec-nano Micro-volume UV-Vis Spectrophotometer (Shimadzu, Kyoto, Japan), and the purified total RNA was stored in deep freeze until further use. Next, total RNA polyadenylation and first-strand cDNA synthesis using an oligo-dT adapter primer were performed, as previously described in detail [27].

### 2.5. Determination of the *IGHV* Mutational Status

PCR amplification and sequencing-based analysis of *IGHV*-*IGHD*-*IGHJ* gene rearrangements were performed as described in Supplementary Materials and Methods. *IGHV* genomic sequences were considered to be mutated when the homology with the closest germ line counterpart was less than 98%, as previously described [26].

### 2.6. RT-qPCR

The developed RT-qPCR methodology exploited the SYBR Green chemistry and was applied in a 7500 Fast Real-Time PCR System (Applied Biosystems, Foster City, CA, USA). Based on the annotated sequences of mature miR-155-5p and *SNORD48* (also known as *RNU48* or *U48*), we designed two specific forward primers which are used along with a common reverse primer to generate two amplicons (68 and 105 bp, resp.). Primer sequences and the detailed RT-qPCR protocol are presented in Supplementary Materials and Methods.

The relative miR-155-5p expression was determined using the comparative C_T_ (2^−ΔΔCt^) method [28, 29], and its prerequisites were checked in a validation experiment. The normalized miR-155-5p expression of each sample was calculated as the ratio of miR-155-5p molecules to *SNORD48* molecules, divided by the same ratio that had been previously calculated for the calibrator (U-937 cells), and was measured in relative quantification units (RQU).

### 2.7. Biostatistical Analysis

The distributions of miR-155-5p expression values in B-CLL patients and normal individuals were not Gaussian; hence, the nonparametric Mann–Whitney *U* test was used to check whether there was a statistically significant difference between the distributions of the two cohorts. We also assessed the diagnostic potential of miR-155-5p expression by receiver operating characteristic (ROC) analysis. Thus, a ROC curve was built by plotting sensitivity versus 1 − specificity, and the respective area under the curve (AUC) was analyzed by the Hanley and McNeil method. Logistic regression was also carried out for the prediction of B-CLL occurrence.

For the determination of the best cut-off value and subsequent categorization of patients into miR-155-5p-positive and miR-155-5p-negative groups, we used the X-tile software [30]. This cut-off point was 1.84 RQU, equal to the 77th percentile. Associations between miR-155-5p expression status and other categorical clinicopathological variables were assessed with the chi-square test or the Fisher exact test, where appropriate.

Kaplan-Meier overall survival (OS) analysis was performed, and differences between OS curves were evaluated using the Mantel-Cox (log-rank) test. Moreover, Cox proportional hazard regression models were built for the assessment of the association between the prognostic markers and the relative risk of succumbing to CLL. The reliability of the developed Cox proportional hazard regression models was strengthened by carrying out bootstrapping in Cox regression analysis, using a number of 2000 samples as well as bias-corrected and accelerated (BCa) 95% confidence intervals. The multivariate Cox regression models were adjusted for the CD38 expression status, presence of *IGHV* mutations, and either clinical stage (Binet or Rai) or risk group. Stratification of B-CLL patients participating in the current study into distinct subgroups with similar prognosis based on established prognostic factors followed by Kaplan-Meier OS analysis was also performed. The level of statistical significance was set at a probability value of less than 0.050 (*p* < 0.050), regarding all statistical tests.

## 3. Results

### 3.1. Biological and Clinical Characteristics of CLL Patients

The group of patients consisted of 62 men and 26 women, with a median age of 70 years (range: 50–90 years) at the time of diagnosis. Using the Binet staging classification, 50 CLL patients (56.8%) were at stage A, 16 (18.2%) at stage B, and 22 (25.0%) at stage C. Positive CD38 expression was encountered in 15 (17.0%) cases, while 73 (83.0%) patients had CD38-negative leukemic B cells. In addition, the early apoptosis index of clonal B cells varied between 0.01 and 54.8 (median value: 4.7), and *IGHV* mutations were detected in 45 (51.1%) CLL cases, whereas 43 (48.9%) patients had wild-type *IGHV*. Biological and clinical features of the CLL patients are presented in Table 1.

### 3.2. miR-155-5p Expression in PBMCs of CLL Patients and Normal Individuals

**Figure 1:**
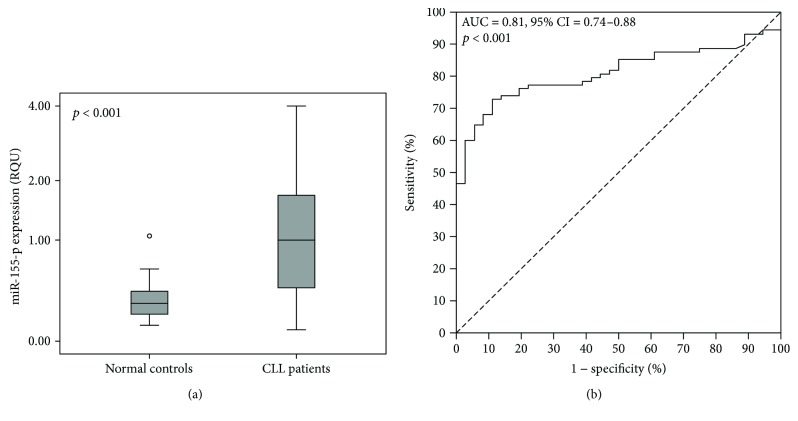
Significant differences are observed in miR-155-5p expression between CLL patients and nonleukemic individuals, thus allowing the discrimination of CLL patients from normal population. (a) Boxplots show the miR-155-5p expression levels in nonleukemic population and CLL patients. The line bars indicate the median value (50th percentile) for each patient cohort, the bottom and top of each box represent the 25th and 75th percentiles, respectively, and the whiskers extend to 1.5 times the height of each box. The *p* value was calculated using the Mann–Whitney *U* test. (b) The ROC curve suggests that miR-155-5p expression could be used as a surrogate diagnostic biomarker in CLL.

In leukemic cells, miR-155-5p expression ranged from 0.081 to 3.99 RQU with a mean ± SE of 1.22 ± 0.11, while in PBMCs of normal samples, the levels of this miRNA varied between 0.11 and 1.06 RQU with a mean ± SE of 0.33 ± 0.030 (Figure 1(a)); this difference was statistically significant (*p* < 0.001). On the other hand, miR-155-5p expression was not associated with CLL patients' age, white blood cell count, lymphocyte count, CD38 expression, or early apoptosis index.

Univariate logistic regression analysis revealed that high miR-155-5p expression could predict CLL occurrence (crude odds ratio = 28.37, 95% CI = 5.20–154.82, *p* < 0.001). More interestingly, ROC analysis showed that miR-155-5p expression can clearly distinguish CLL patients from normal controls (area under the curve (AUC) = 0.81, 95% confidence interval (95% CI) = 0.74–0.88, *p* < 0.001), as also illustrated in Figure 1(b).

### 3.3. Independent Unfavorable Prognostic Value of miR-155-5p Overexpression in CLL Patients

Based on the optimal prognostic cut-off point that was determined for miR-155-5p expression values, as described in detail in Materials and Methods, 68 (77.2%) CLL cases were categorized as miR-155-5p-negative and 20 (22.7%) as miR-155-5p-positive.

**Figure 2:**
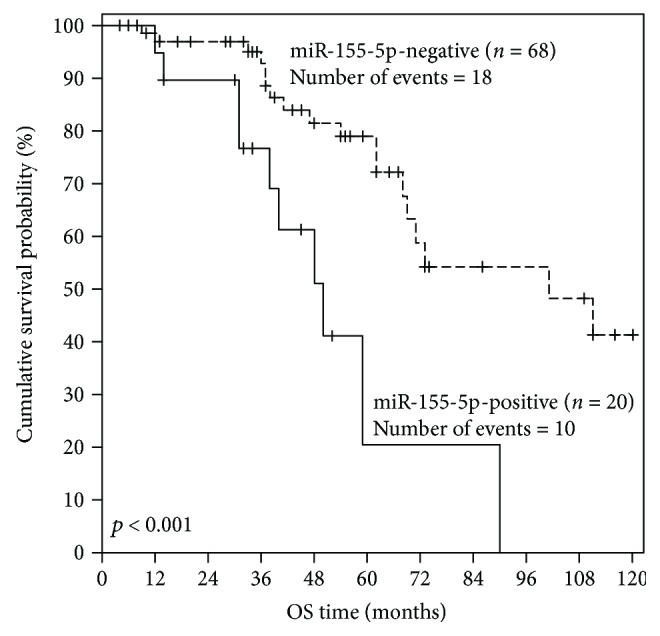
Kaplan-Meier OS curves for CLL patients stratified according to miR-155-5p expression in their PBMCs. miR-155-5p expression status is a significant prognostic factor in CLL, as miR-155-5p-positive patients have significantly inferior OS, compared to miR-155-5p-negative patients. The *p* value was calculated using the Mantel-Cox (log-rank) test.

Follow-up information was available for all patients, 28 (31.8%) of whom died during the accrual period due to causes related to CLL. The estimated median OS was 73 months (95% CI = 45–101 months). Kaplan-Meier analysis revealed significantly reduced OS for miR-155-5p-positive CLL patients in comparison with miR-155-5p-negative patients (*p* < 0.001) (Figure 2). In agreement with these results, univariate Cox regression analysis uncovered a significant (*p* = 0.001), quite 4-fold higher risk of death for CLL patients exhibiting miR-155-5p positivity (Table 2). Bootstrap Cox regression models highlighted the robustness of the conclusion that miR-155-5p overexpression is a significant unfavorable prognosticator in CLL (*p* < 0.001) (Table 2).

**Figure 3:**
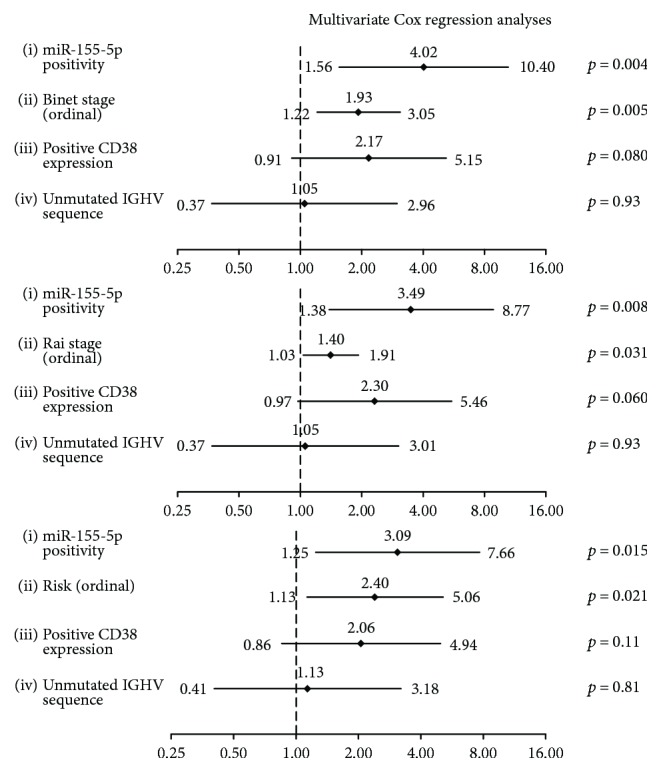
Forest plots showing multivariate Cox regression analyses of the effect of miR-155-5p expression status and clinicopathological and biological variables on the OS of CLL patients. The multivariate models were adjusted for the CD38 expression status, presence of *IGHV* mutations, and either clinical stage (Binet or Rai) or risk group. These two staging systems share common components (enlarged lymph node areas, anemia, and low levels of platelets), and risk classification is based on Rai staging; consequently, these three prognosticators cannot be included in the same multivariate Cox regression model. Diamonds indicate the hazard ratios, and horizontal lines represent the 95% confidence interval of each hazard ratio.

Most importantly, the unfavorable prognostic significance of miR-155-5p overexpression was shown to be independent of CD38 expression, unmutated *IGHV*, and the clinical stage (Binet or Rai stage) or risk group, as demonstrated in the multivariate Cox regression analysis and illustrated by the respective forest plots (Figure 3).

### 3.4. Unfavorable Prognostic Value of miR-155-5p Overexpression in Distinct Subgroups of CLL Patients, Stratified According to Established Prognostic Factors

**Figure 4:**
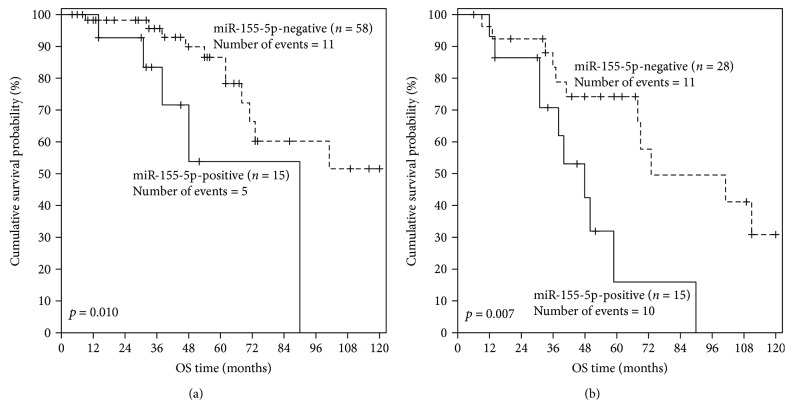
Kaplan-Meier curves for the OS of subgroups of CLL patients with different prognoses. miR-155-5p positivity was shown to have a significant unfavorable prognostic value for CLL patients with (a) negative CD38 expression (≤30%) and/or (b) unmutated *IGHV* genomic sequence.

As the prognosis of patients with negative CD38 expression and/or unmutated *IGHV* genomic sequences is substantially different from the prognosis of patients showing positive CD38 expression and/or mutated *IGHV*, Kaplan-Meier OS analysis was performed in substantially different prognostic subgroups of CLL patients, stratified according to each one of these established prognostic factors, in order to evaluate the potential additional prognostic value of miR-155-5p expression. As clearly illustrated in Figure 4(a), miR-155-5p positivity predicts an unfavorable outcome also among CLL patients with negative CD38 expression (*p* = 0.010). Similarly, CLL patients with unmutated *IGHV* sequence combined with strong miR-155-5p expression had significantly lower OS probabilities than those with unmutated *IGHV* and negative miR-155-5p expression status (*p* = 0.007) (Figure 4(b)).

## 4. Discussion

miRNAs play a major role in almost every normal cellular process in animals, including normal hematopoiesis. Dysregulation of their expression levels contributes significantly to the development and/or progression of many human diseases, including cancer and leukemias [31]. Several miRNAs have been suggested as promising therapeutic targets in the battle against leukemia, as restoration of their normal expression in leukemic cells can dramatically alter the expression levels of key proteins that are heavily involved in leukemia pathobiology [32]. Moreover, some of these small noncoding RNAs have prognostic value in particular hematological malignancies [32].

miR-155-5p constitutes an important miRNA in lymphoid differentiation. High levels of miR-155-5p are present in activated B and T cells and in activated monocytes [33]. miR-155-5p tunes finely germinal center reaction and T helper cell differentiation by affecting cytokine production [34]. Furthermore, miR-155-5p controls the function of lymphocytes and dendritic cells and is critical for normal immune function [35]. Of note, miR-155-5p targets forkhead box 3 (FOXO3) [36], a transcription factor acting downstream of the PTEN/PI3K/AKT pathway and being indispensable for self-renewal of hematopoietic stem cells [37]. miR-155-5p expression is upregulated in AML patients with FLT3-ITD mutations [38–41]. On the other hand, sustained expression of miR-155-5p is associated with enhanced myeloid proliferation [42]. Thus, miR-155-5p seems to play a significant role in the pathogenesis of a particular subgroup of AML patients [41]. Very recently, the dysregulation of the miR-155-5p/miR-150 network has been linked to CLL pathogenesis, as it attenuates the expression of the *PU.1* proto-oncogene, a well-known regulator of differentiation in B cells [43]. Moreover, strong miR-155-5p expression was observed in CLL proliferation centers [44]. Nonetheless, the prognostic potential of miR-155-5p expression in CLL has not been studied so far.

To the best of our knowledge, this is the first research study showing that miR-155-5p expression may possess diagnostic value regarding CLL, as miR-155-5p levels are significantly higher in PBMCs of CLL patients, compared to PBMCs of nonleukemic controls. On the other hand, no significant associations were found between miR-155-5p overexpression and CLL patients' clinicopathological features, including Binet stage, Rai stage, risk groups, serum LDH concentration, and cell apoptotic index. However, based on previous results according to which there is a progressive increase in miR-155-5p expression from control cases to patients with monoclonal B cell lymphocytosis and to those with early-stage CLL [45], we decided to examine the putative prognostic value of miR-155-5p expression in CLL.

Although a lot of scientific efforts have been put on the discovery of novel prognostic biomarkers in CLL during the last two decades, only a few of them have led to the establishment of such prognostic biomarkers in the clinics [46]. Staging of the disease, mutations of the *IGHV* genomic sequences, and loss or mutation of the *TP53* gene are probably the most valid prognosticators in CLL [47, 48]. As it is occasionally rather hard to determine the mutational status of the *IGHV* gene, several studies sought to identify new promising surrogate markers, such as ZAP70 and CD38 [49, 50]. Other significant molecular prognostic biomarkers include particular mutations in *NOTCH1* [51] and splicing factor 3b subunit 1 (*SF3B1*) genes [52] and chromosomal abnormalities such as deletions of 11q, 13q, 17p, and trisomy 12 [53, 54]. In the current study, *IGHV* mutational status and CD38 expression were verified as significant predictors of prognosis, in contrast to high serum LDH concentration, a typical feature of Richter's transformation that can arise in CLL patients [55]. The mutational status of *TP53*, *SF3B1*, and *NOTCH1* genes and the aforementioned chromosomal abnormalities were not examined.

Regarding the prognostic value of miR-155-5p expression in CLL, Kaplan-Meier OS analysis revealed significantly lower survival rates for miR-155-5p-positive patients. Moreover, univariate Cox regression analysis indicated that high miR-155-5p expression in PBMCs of CLL patients predicts an increased risk of death. Importantly, the high prognostic significance of miR-155-5p positivity is independent of clinical staging and other prognostic markers, including *IGHV* mutational status and CD38 expression. Interestingly, according to the developed multivariate Cox regression models, the prognostic significance of miR-155-5p expression status is independent of CD38 expression, unmutated *IGHV*, and the clinical stage (Binet or Rai) or risk category, which represent some of the most valid prognostic markers in CLL. There is no doubt that the prognostic value of miR-155-5p expression needs to be further validated in larger and independent cohorts of CLL patients. However, the bootstrapping that was performed in Cox regression strengthens the hypothesis that miR-155-5p overexpression is a promising molecular biomarker of unfavorable prognosis in CLL. Therefore, a panel of molecular prognostic biomarkers that have recently emerged—e.g., the human leukocyte antigen (HLA-G) [56], high histone deacetylase (HDAC) activity [57], mRNA expression of kallikrein-related peptidase 14 (*KLK14*) [58] and BCL2-like 12 (*BCL2L12*) [59], circulating miR-150 [60], SRY-box 1 (SOX1) [61], and serum soluble transmembrane activator calcium modulator and cyclophilin ligand interactor (TACI) [62]—could prove even more promising, provided that it integrates miR-155-5p expression as a new component. In support of this suggestion, it should be added that accumulation of several unfavorable prognostic markers has been proven to deteriorate prognosis in CLL [63]. Moreover, it would be tempting to examine the combinatorial value of all these previously suggested prognostic biomarkers besides miR-155-5p expression in a large multicenter study, in order to generate a prognostic score in CLL, like the one that has recently been proposed by the German Chronic Lymphocytic Leukemia Study Group [64].

In conclusion, we developed a cost-effective, sensitive, and accurate SYBR Green-based qPCR technique for the quantification of miR-155-5p levels in PBMCs and investigated its putative discriminatory and prognostic value in CLL. It should be added that the establishment of an optimal cut-off point and the subsequent categorization of miR-155-5p expression values as positive or negative alleviate the need for utmost sensitivity that could be offered by a ready-to-use qPCR assay using TaqMan probes, rendering such commercially available assays less cost-effective [65]. Our study showed that high miR-155-5p expression represents a novel, potential molecular biomarker of unfavorable prognosis in CLL, meriting further investigation in a large cohort of CLL patients. Ideally, miR-155-5p expression status could be combined with surrogate prognostic biomarkers in a multiparametric prognostic index with regard to this hematological malignancy, as the high prognostic significance of miR-155-5p overexpression is independent of clinical staging and other already established biomarkers (*IGHV* mutational status and CD38 expression) that are used to predict CLL patients' outcome.

## Figures and Tables

**Table 1:** Biological and clinicopathological features of CLL patients.

Total number of patients	88
Patients' sex (male/female)	62/26
	Median (range)
Age (years)	70 (50–90)
OS (months)	42 (4–120)
White blood cells (×10^6^/mL)	32,250 (8500–96,900)
Lymphocytes (×10^6^/mL)	23,050 (5300–69,430)
CD38 expression (mean optical intensity of staining)	7.7 (0.10–59.2)
Early apoptosis index (55/88 patients)	4.7 (0.01–54.8)
	Number of patients (%)
Binet stage	
A	50 (56.8%)
B	16 (18.2%)
C	22 (25.0%)
Rai stage	
0	16 (18.2%)
I	27 (30.7%)
II	24 (27.3%)
III	4 (4.5%)
IV	17 (19.3%)
Risk group	
Low	16 (18.2%)
Intermediate	51 (58.0%)
High	21 (23.9%)
Serum LDH concentration	
Normal	60 (68.2%)
Elevated	28 (31.8%)
CD38 expression status	
Negative	73 (83.0%)
Positive	15 (17.0%)
*IGHV* mutational status	
Mutated	45 (51.1%)
Unmutated	43 (48.9%)

CLL: chronic lymphocytic leukemia; OS: overall survival; LDH: lactate dehydrogenase; *IGHV*: immunoglobulin heavy chain variable region.

**Table 2:** Cox proportional hazard univariate regression analysis of miR-155-5p expression and clinicopathological variables for the prediction of CLL patients' OS.

Covariate	HR	95% CI	*p* value	BCa bootstrap (95% CI)	Bootstrap (*p* value)
miR-155-5p expression	1.80	1.19–2.72	0.006	1.08–3.00	0.007
miR-155-5p expression status					
Negative	1.00				
Positive	3.87	1.70–8.81	0.001	1.76–11.72	<0.001
Age	0.98	0.94–1.02	0.33	0.93–1.02	0.36
Binet stage (ordinal)	1.98	1.3–3.02	0.002	1.38–2.97	<0.001
Rai stage (ordinal)	1.50	1.14–1.98	0.004	1.16–2.02	0.002
Risk (ordinal)	3.01	1.52–5.94	0.002	1.85–5.29	<0.001
Serum LDH concentration					
Normal	1.00				
Elevated	1.61	0.76–3.41	0.22	0.74–3.66	0.21
CD38 expression status					
Negative	1.00				
Positive	3.57	1.68–7.58	<0.001	1.46–13.11	<0.001
*IGHV* mutational status					
Mutated	1.00				
Unmutated	2.78	1.18–6.55	0.020	1.19–7.99	0.013

CLL: chronic lymphocytic leukemia; OS: overall survival; HR: hazard ratio; CI: confidence interval; BCa: bias-corrected and accelerated; LDH: lactate dehydrogenase; *IGHV*: immunoglobulin heavy chain variable region.
