# Supplementary material for: MicroRNA-155-5p Overexpression in Peripheral Blood Mononuclear Cells of Chronic Lymphocytic Leukemia Patients Is a Novel, Independent Molecular Biomarker of Poor Prognosis
Source: Dis Markers. 2017 Dec 31;2017:2046545. doi: 10.1155/2017/2046545 (PMC5804407; doi:10.1155/2017/2046545)
Supplement: Supplementary Materials — and methods. [file 2046545.f1.pdf]

Supplementary Materials and Methods

Cell immunophenotyping. Immunophenotyping of B-CLL cells was performed with a routine panel of evaluated monoclonal antibodies such as anti-CD3, -CD5, -CD10, -CD11c, -CD19, -CD20, -CD23, -FMC7, -Igκ, and –Igλ (Immunotech, Prague, Czech Republic), conjugated with fluorescein isothiocyanate (FITC), R-phycoerythrin (R-PE), or cyanine-5 (Cy5), with flow cytometry in a Coulter® Epics® XL-MCL™ Flow Cytometer (Beckman Coulter Inc., Miami, FL, USA). B-CLL diagnosis was established by detecting the leukemic $CD5^+/CD19^+/CD23^+$ cell clone. A monoclonal anti-CD38 antibody was used for CD38 expression analysis.

Early apoptosis detection. For whole blood lysis, 2 mL of whole blood were mixed with ammonium chloride in a proportion 1:7. After centrifugation, the supernatant was discarded and the remaining cell pellet was washed three times with PBS (pH 7.2, 25°C). Cells were co-stained with Annexin V-FITC (green fluorescence signal) and propidium iodide (PI), thus allowing the simultaneous discrimination of leukemic cells into intact (FITC–/PI–), early apoptotic (FITC+/PI–), and late apoptotic or necrotic ones (FITC+/PI+); thus, the percentage of early apoptotic leukemic cells (early apoptosis index) was determined. Briefly, 1 μL of Annexin V-FITC (Immunotech) solution and 5 μL of PI (Immunotech) were added in the resuspended cell pellet and gently mixed, followed by addition of 20 μL of anti-CD19-PE (phycoerythrin; Immunotech) antibody. The tubes were incubated in the dark, on ice, for 15 minutes. Next, 400 μL of ice-cold binding buffer were added and the cell preparation was analyzed by two-color flow cytometry in a Coulter® Epics® XL-MCL™ Flow Cytometer (Beckman Coulter Inc.). At the FS/SS plot, the $CD19^+$ cell population (B cells) was further analyzed.

Determination the IGHV mutational status. Clonal PCR products were purified with the QIAquick Gel Extraction Kit (Qiagen Inc., Valencia, CA, USA) and both strands were sequenced, based on fluorescence dideoxy chain termination, in a CEQ™ 8000 Genetic Analysis System (Beckman Coulter Inc.). Sequencing data were analyzed using IMGT®, the International ImMunoGeneTics information system® (http://imgt.cines.fr) and, more particularly, the IMGT/V-QUEST and IMGT/JunctionAnalysis tools.

RT-qPCR. SNORD48 was chosen as reference for the normalization of qPCR for the RNA quantity added to the reverse transcription. Moreover, cDNA prepared from RNA of U-937 cells was used as calibrator to render all data obtained by distinct qPCR runs comparable.

The sequence of the miR-155-5p forward primer was 5'-TTAATGCTAATCGTGATAGGGGTTAA-3' and that of the *SNORD48* forward primer was 5'-TGATGATGACCCCAGGTAACTCT-3', while the sequence of the common reverse primer binding to the oligo-dT adapter was 5'-GCGAGCACAGAATTAATACGAC-3'. The PCR amplicons for miR-155-5p and *SNORD48* were 68 and 105 long, respectively.

The reaction mixture contained 1 μL of 10-fold diluted cDNA, 5 μL KAPA™ SYBR® FAST qPCR master mix (2X) (Kapa Biosystems Inc., Woburn, MA, USA), and 2 μL of primers (final concentration: 300 nM each), in a final reaction volume of 10 μL. The following cycling conditions were used: a denaturation step at 95 °C for 3 min, 40 cycles of 95 °C for 3 sec, for denaturation of the PCR products, and 60 °C for 30 sec, for primer annealing and extension as well as for detection of fluorescence. Melting curves of the PCR products were generated by heating the reaction from 60 °C to 95 °C with a rate of 0.3 °C/sec and continuously acquiring fluorescence emission data, so as to distinguish the specific PCR products from non-specific products, characterized by a different T_m than those of the miR-155-5p and *SNORD48* amplicons. All reactions were performed in duplicate.
